# Accuracy of glomerular filtration rate estimates among patients with cancer

**DOI:** 10.1038/s41416-025-03190-3

**Published:** 2025-09-19

**Authors:** Jennifer S. Lees, Edouard L. Fu, Anne-Laure Faucon, Benjamin MP Elyan, Lesley A. Inker, Andrew S. Levey, Robert J. Jones, Richard H. Wilson, Patrick B. Mark, Juan-Jesus Carrero

**Affiliations:** 1 School of Cardiovascular and Metabolic Health, University of Glasgow, Glasgow, United Kingdom; 2 NHS Greater Glasgow and Clyde, Glasgow, United Kingdom; 3 Department of Clinical Epidemiology, Leiden University Medical Center, Leiden, The Netherlands; 4 Department of Medical Epidemiology and Biostatistics, Karolinska Institutet, Stockholm, Sweden; 5 Division of Nephrology, Department of Medicine, Tufts Medical Center, Boston, Massachusetts, United States of America; 6 School of Cancer Sciences, University of Glasgow, Glasgow, United Kingdom; 7 Division of Nephrology, Department of Clinical Sciences, Karolinska Institutet, Danderyd Hospital, Stockholm, Sweden

**Keywords:** Biomarkers, Kidney diseases, Cancer

## Abstract

**Background:**

Glomerular filtration rate (GFR) estimation is a key issue in determining cancer treatment eligibility and dosing of treatments with narrow therapeutic index. Yet, little is known about the accuracy of GFR estimation among people with cancer in routine care.

**Methods:**

In a cross-sectional study including 1611 adults with cancer referred for 1837 determinations of measured GFR (mGFR), we assessed the accuracy of estimated GFR based on creatinine (eGFRcr), cystatin C (eGFRcys) and their combination (eGFRcr-cys). Accuracy was reported as percentage of patients with estimated values within 30% of mGFR; bias and precision as the median and interquartile range of eGFR-mGFR, respectively. Dosing accuracy was assessed by calculating expected dose of carboplatin for area under the curve of 5 mg/mL/min using the Calvert formula.

**Results:**

Median age was 68 (IQI 61 to 74) years, 38.5% were female with mean mGFR 75 (SD 30) mL/min; 17% had metastatic disease. Accuracy, bias and precision were best for eGFRcr-cys. Using eGFRcr would recommend an “overdose” of carboplatin in 10–20% of participants: this was 3–4 times less common using eGFRcr-cys.

**Conclusion:**

eGFRcr-cys equations provide the most accurate estimates of mGFR in patients with cancer, with potential to improve dosing accuracy substantially compared to eGFRcr.

## Introduction

Accuracy of glomerular filtration rate (GFR) estimation is a key issue in determining cancer treatment eligibility and dosing of treatments with narrow therapeutic index. Many anti-cancer drugs, including most cytotoxics, are administered near the maximum tolerated dose and have a narrow therapeutic index [1]. For medications excreted by the kidney (e.g. carboplatin), dosing requires accurate GFR assessment, even in people who are considered to have normal GFR. For medications that are not primarily excreted by the kidney (e.g., monoclonal antibodies like trastuzumab [Herceptin]), GFR may still influence pharmacokinetics due to altered hepatic metabolism—increasingly common with worsening GFR [2]—and removal of inactive but potentially toxic metabolites. Errors in GFR estimation may expose people with cancer to inappropriate exclusion or inclusion for systemic anti-cancer therapy, under-dosing and under-treatment, or over-dosing and treatment-associated harms [3, 4]. Yet, little is known about the accuracy of GFR estimation among people with cancer in routine care.

The gold standard method to assess GFR is a urinary or plasma clearance of an exogenous filtration marker, such as iohexol or markers linked to radionucleotides, referred to as measured GFR (mGFR); however, these methods are costly and/or not available in all hospitals, limiting their routine use in the management of patients with cancer. In most cases, GFR is instead estimated using creatinine: a cheap and widely available endogenous filtration marker which is generated as the end-product of creatine metabolism in skeletal muscle. Creatinine may be measured in the blood and incorporated into estimation equations, but discrepancies between estimated and measured GFR are common, can be large, may be greater among patients with cancer than in the general population, and primarily reflect limitations of creatinine as a filtration marker.

Creatinine levels are altered by factors other than GFR, such as changes in muscle mass and activity, protein intake and tubular secretion (particularly with co-administration of other medications): all commonly disturbed in people with cancer. In general population settings, diagnosis of CKD, identification of risk and treatment allocation may be improved by additionally testing serum cystatin C. Cystatin C is a low-molecular-weight protein produced by all nucleated cells and its non-GFR determinants (such as smoking, adiposity, inflammation and glucocorticoid treatment [5]) differ from those of creatinine [6–8]. In combination with creatinine, cystatin C (eGFRcr-cys) improves accuracy of GFR estimation (and therefore dosing accuracy) in the general population as well as populations with cancer [3, 9–12]. Kidney Disease Improving Global Outcomes (KDIGO) international guidance for CKD evaluation and management recommends use of eGFRcr-cys where eGFRcr is less accurate and GFR affects clinical decision-making: cancer is listed specifically as a condition where this is indicated [13]. Based on a systematic review of the performance of eGFR equations in patients with cancer, the American Society of Onco-Nephrology issued a position statement in 2024 advocating the use of eGFR equations incorporating both creatinine and cystatin C in patients with cancer [14]. The evidence was considered moderate to low quality, the recommendation was graded as low certainty, and only one study of 112 patients in routine care testing utility of cystatin C was included in the review. The authors called for additional studies assessing the accuracy of eGFR equations in patients with cancer. Due to additional costs, lack of widespread availability and concerns over non-GFR determinants of cystatin C, data to support the generalisability of these findings to routine care settings are lacking.

We aimed to assess the accuracy of currently available eGFR equations using creatinine, cystatin C or both filtration markers, against mGFR, in patients with cancer managed in routine care, and to analyse potential implications for treatment eligibility and dosing.

## Methods

### Data source

Participants were from the Stockholm CREAtinine Measurements Project (SCREAM), a complete healthcare utilization cohort of all citizens from Region Stockholm, Sweden, during 2006-2021 [15]. SCREAM uses unique personal identification numbers to integrate regional and national administrative databases containing comprehensive data on demographics, healthcare utilisation, prescribed medications, diagnoses, vital status, kidney replacement therapy and completed laboratory tests [15]. SCREAM has been linked to the National Cancer Register, which collates compulsory reports of new cancer diagnoses from all public and private healthcare providers, and details the date of cancer diagnosis, tumour site, stage, behaviour (benign or malignant), histological type and basis of diagnosis (e.g., clinical or pathological) [16]. SCREAM was approved by the Stockholm Ethics Review Board with a waiver of consent (reference 2017/793-31).

### Study design

This was a cross-sectional study of adults (age 18 years or older) who had a diagnosis of cancer between 1^st^ January 2010 and 31^st^ December 2021; with a determination of measured glomerular filtration rate (mGFR) using single-point plasma clearance of iohexol within 2 years after a diagnosis of cancer; with concurrent measurements of creatinine and cystatin C taken on the same day and within 30 days of mGFR; and with available height and weight data within 30 days of mGFR (see “study covariates” below). The date of mGFR was considered the index date. After applying eligibility criteria, there were 1611 adults with cancer with 1837 determinations of mGFR and concurrent creatinine and cystatin C.

### Cancer diagnosis

Cancer diagnosis was identified from the Swedish Cancer Register, International Classification of Disease, 10^th^ Edition (ICD-10) codes C00-C97, excluding C44 (non-melanoma skin cancer). From 1^st^ January 2010 to 31^st^ December 2021, there were 116,627 individuals with 128,813 unique diagnoses of cancer. Cancer types were defined by ICD-10 codes (Supplementary Table S1). Staging information at the time of cancer diagnosis was available from used to identify “metastatic” cancer, defined as a recorded stage “IV” in International Federation of Gynaecology and Obstetrics [FIGO] classification for vulval cancers or stage “M1” in Tumour Node Metastasis (TNM) Classification of Malignant Tumours for all other cancers where staging information was available; otherwise, cancers were classified as “not metastatic”. Where staging information was not available (including for all cancers of the brain, cranial nerves, lymphoma and leukaemia), cancers were classified as having “unknown” presence of metastatic disease.

### GFR measurement

Usual practice in Stockholm health care would be to measure iohexol clearance after cancer diagnosis, in patients who were likely to be treated with systemic anti-cancer therapies, which may include repeated measures over time. Iohexol clearance was measured using single-point plasma clearance of iohexol at the central laboratory, Department of Clinical Chemistry, Karolinska University Hospital in Stockholm, following systematic protocols [17]. In brief, 5 mL of iohexol (omnipaque 300 mg/mL, GE Healthcare) was administered by intravenous injection, and then a 10 mL normal saline flush was performed. Plasma clearance measurement was obtained from 5 mL blood samples from the contralateral arm to the injection. Timing of sample collection was based on eGFR; samples were collected after approximately 4 hours for eGFRcr >40 mL/min/1.73 m^2^, 6-8 hours for eGFRcr 15-40 mL/min/1.73 m^2^, 24 hours for eGFRcr <15 mL/min/1.73 m^2^. Precise times of injection and sample collection were recorded. When transport of samples to central laboratories could not take place on the same day, samples were centrifuged prior to transport. Ultra-high performance liquid chromatography separation and ultraviolet detection were used to determine serum iohexol concentration. Internal controls were used to monitor the performance of the iohexol method, and the Government-run monitoring company Equalis (Uppsala, Sweden) verified the performance of the iohexol method through an external quality assurance program for country-wide iohexol standardization. Values were indexed to body surface area (BSA; indexed mGFR) and reported in mL/min/1.73m^2^. For this analysis, values were converted to non-indexed mGFR using BSA calculated from height and weight according to the Du Bois and Du Bois formula [18] and reported in mL/min (see “study covariates” below). Implausible values of <0 or >150 mL/min were excluded.

### GFR estimating equations

Although methods and/or analysers have changed over time, during the interval of our study, creatinine values were standardised to isotope dilution mass spectrometry (IDMS) traceable methods [19], and cystatin C was traceable to International Federation of Clinical Chemistry and Laboratory Medicine (IFCC) reference materials [20]. We calculated eGFR based on serum or plasma values of creatinine (eGFRcr), cystatin C (eGFRcys) or the combination of both filtration markers (eGFRcr-cys) using 13 equations. For the main paper, we include the Chronic Kidney Disease Epidemiology Collaboration (CKD-EPI) and European Kidney Function Consortium (EKFC) equations:

- eGFRcr: CKD-EPI 2009 [21]; EKFC 2021 [22]
- eGFRcys: CKD-EPI 2012 [9]; EKFC 2023 [22]
- eGFRcr-cys: CKD-EPI 2012 [9]; EKFC 2023 (the arithmetic mean of eGFRcr EKFC 2021 and eGFRcys EKFC 2023)

Details of the selection of the 6 equations for the main paper and the additional 7 equations shown in the Supplementary Data File are provided in the Supplementary Methods. Derivation code for each equation will be provided in the project GitHub page on publication of the results (https://github.com/jennifer-s-lees/scream_cancer_egfr_accuracy_public).

For the primary analysis, indexed eGFR values (in mL/min/1.73m^2^) were converted to non-indexed eGFR (as above for mGFR) and reported in mL/min to ensure comparability between eGFR and mGFR and to optimise accuracy for drug dosing calculations. In a sensitivity analysis, we additionally report bias (see “statistical analysis” below) for eGFR compared to mGFR with both non-indexed and indexed to BSA (in mL/min/1.73m^2^). Of note, bias for indexed eGFR vs. mGFR differs minimally from bias for non-indexed eGFR vs. mGFR except for individuals with very small or very large BSA [23].

### Study covariates

Height and weight data were preferentially extracted on the index date (i.e., the date of mGFR), enriched with measurements collected +/- 30 days from the index date. Where height and weight data were not available (28.1% of the cohort with mGFR and concurrent creatinine and cystatin C), participants were excluded from further analysis. Compared with the included cohort, those with missing height and weight data were younger, less often female, with lower rates of metastatic disease, pre-existing diabetes, cardiovascular disease, hypertension and liver disease. Those with missing height and weight data also had similar mGFR indexed to BSA (71 vs. 68 mL/min/1.73m^2^, p = 0.06; Supplementary Table S2).

Body mass index (BMI) was calculated using the formula: BMI = (weight in kg) / (height in m)^2^. BSA was calculated from height and weight using the Du Bois and Du Bois formula: BSA = 0.007184 * (height in cm)^0.725^ * (weight in kg)^0.425^.

Comorbid conditions were defined by the presence of relevant ICD-10 codes prior to the index date (Supplementary Table S3). Steroid use was defined using Anatomical Therapeutic Chemical classification H02AB (systemic glucocorticoids). Steroid use was assumed to be concomitant if there was a recorded dispensation from pharmacy in the nationwide prescribed drugs register, within 6 months prior to the index date.

### Statistical analysis

Baseline characteristics were summarised as mean (standard deviation; SD), median (interquartile interval; IQI) or number (percentage), as appropriate. Results are presented for the overall group in the main paper and stratified by sex in the data supplement.

Accuracy was reported as the percentage of patients with estimated values that were within 30% of mGFR (P30) and 15% of mGFR (P15) [13]. For general use, a P30 value of <80% is considered unacceptable, 80%–90% is considered acceptable, and values of >90% are considered optimal [13]. Though there are no formal targets recommended for the treatment of cancer, safe dosing of medications with narrow therapeutic indices warrants aiming for the highest possible precision; for mGFR this corresponds approximately to P15 of 90% [24, 25]. Of note, P30 and P15 for indexed eGFR versus mGFR have been shown previously to be identical to P30 and P15 for non-indexed eGFR versus mGFR [23].

Agreement was assessed using Bland-Altman plots. Bias was estimated as median difference of eGFR minus mGFR. Small, medium, and large bias are defined as ±5, ±5 to ±10, and more than ±10 mL/min. Small bias (within ±5 mL/min) can be due to differences in measurement methods for creatinine, cystatin C and mGFR. Precision was estimated as the interquartile range (IQR) of the bias.

The most implemented treatment eligibility thresholds seen in recent phase 3 trials of combination cancer therapies range from 30-60 mL/min [26]. Contingency tables were created to assess the agreement across eGFR versus mGFR categories of <30, 30-44, 45-59, 60-89, 90-119, and >120 mL/min. Correct classification was assessed as percent agreement across all categories.

We assessed the potential for dosing inaccuracies of cancer treatments according to equation and filtration marker. Prescribed and/or administered systemic anti-cancer therapies are not currently available through SCREAM. We chose carboplatin as an exemplar because: i) it is a platinum-based chemotherapy excreted primarily by the kidney; ii) it is used in a range of cancer types; iii) it has a narrow therapeutic index; and iv) the dose calculation includes kidney function: carboplatin dose (mg) = target area under the curve [AUC; mg/mL/min] × (GFR [mL/min] + 25], with an upper GFR limit of 125 mL/min. We excluded n = 11 individuals with mGFR <30 mL/min for this analysis, as carboplatin treatment would be contraindicated in these individuals. For mGFR and each eGFR equation, we calculated the recommended dose of carboplatin assuming target AUC of 5 mg/mL/min: a common target in clinical practice, balancing efficacy while minimising haematologic toxicity [24]. For each eGFR equation, we then reported the proportion of individuals where the recommended dose would expect to produce an AUC of more than 6 mg/mL/min (“overdose”; potentially causing treatment-associated harm) or less than 4 mg/mL/min (“underdose”; potentially leading to reduced efficacy) compared to mGFR.

We considered accuracy, precision, bias and dosing inaccuracies in subgroups where sources of error in eGFRcr and/or eGFRcys may be more pronounced: sex (female versus male), BMI category (<20, 20 to <25, 25 to <30, ≥30 kg/m^2^) and metastatic disease (metastatic vs. not metastatic vs. unknown).

Analyses were conducted using *RODBC*, *tidyverse*, *tableone*, *broom* and *ggpubr* packages for R statistical software (version 4.3.1 using RStudio for Windows version 2023.09.0).

## Results

### Population

In 1837 determinations of mGFR, the median age of participants was 68 (IQI 61 to 74) years, 38.5% were female with mean mGFR 75 (SD 30) mL/min (Table 1). Bladder (33%), lung (23%) and colorectal (13%) cancers were most common; 17% had metastatic disease.

**Table 1:** Baseline characteristics of included participants.

	Overall
N (%)	1837
Age: years	68 [61, 74]
Female: n(%)	707 (38.5)
Body mass index: kg/m^2^	25.3 (4.5)
Body surface area: m^**2**^	1.9 (0.2)
Comorbidity count	1.0 [0.0, 2.0]
Cancer type (%)	
Bladder	599 (32.6)
Colorectal	229 (12.5)
Female genital organs	129 (7.0)
Kidney/ureter	120 (6.5)
Lung and bronchus	428 (23.3)
Male genital organs	72 (3.9)
Other	260 (14.2)
Metastatic: n(%)	
Not metastatic	1212 (66.0)
Metastatic	319 (17.4)
Unknown	306 (16.7)
Systemic glucocorticoid use: n(%)	621 (33.8)
Diabetes: n(%)	379 (20.6)
Hypertension: n(%)	950 (51.7)
Cardiovascular disease: n(%)	576 (31.4)
Liver disease: n(%)	232 (12.6)
mGFR: mL/min	75 (30)
eGFRcr CKD-EPI 2009: mL/min	81 (28)
eGFRcr EKFC 2021: mL/min	76 (26)
eGFRcys CKD-EPI 2012: mL/min	67 (31)
eGFRcys EKFC 2023: mL/min	67 (25)
eGFRcr-cys CKD-EPI 2012: mL/min	74 (29)
eGFRcr-cys EKFC 2023: mL/min	71 (25)

Mean (standard deviation; SD) or median (interquartile range; IQR) unless otherwise specified. CKD-EPI: Chronic Kidney Disease Epidemiology Collaboration; eGFRcr: estimated GFR by creatinine; eGFRcys: estimated GFR by cystatin C; eGFRcr-cys: estimated GFR by creatinine and cystatin C; EKFC: European Kidney Function Consortium; mGFR: measured glomerular filtration rate (GFR) by single-point plasma clearance of iohexol.

### P30 and P15

**Fig. 1:**
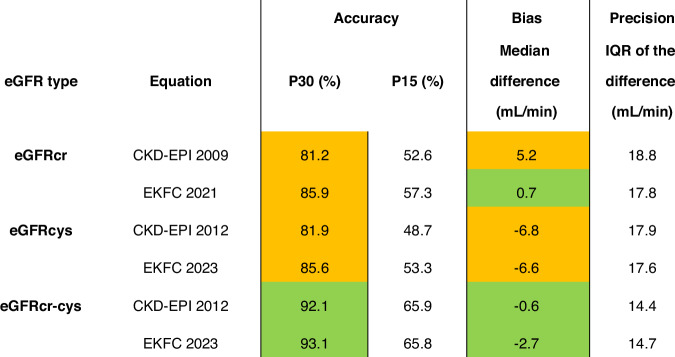
P30, P15 and Bias. Percentage of patients with estimated values that were within 30% of mGFR (P30) and within 15% of mGFR (P15). Bias was estimated as median difference of eClcr or eGFR minus mGFR. IQR: interquartile range of the bias. mGFR: measured glomerular filtration rate (GFR) by single-point iohexol clearance; eClcr: estimated creatinine clearance; eGFRcr: estimated GFR by creatinine; eGFRcys: estimated GFR by cystatin C; eGFRcr-cys: estimated GFR by creatinine and cystatin C; EKFC: European Kidney Function Consortium. Green: optimal accuracy/small bias; Amber: adequate accuracy/medium bias; Red: inadequate accuracy/large bias.

eGFRcr-cys provided optimal P30 values (>90%), whereas eGFRcr and eGFRcys provided acceptable P30 values (80-90%; Fig. 1 and Supplementary Table S4). Similarly, eGFRcr-cys equations demonstrated consistently higher values for P15 than either eGFRcr or eGFRcys equations (Fig. 1 and Supplementary Table S4).

### Agreement, Bias and Precision

Agreement was best for eGFRcr-cys equations (Supplementary Fig. S1). eGFRcr and eGFRcys showed similar levels of agreement, but eGFRcr often overestimated mGFR and eGFRcys systematically underestimated mGFR. Median bias was similar and small for eGFRcr and eGFRcr-cys (Fig. 1); however, precision was best for eGFRcr-cys. Bias and IQR for mGFR and eGFR indexed to BSA were similar to bias and IQR for non-indexed mGFR and eGFR (Supplementary Table S5).

### Correct classification

Correct classification across mGFR categories was best and within the expected range of 60-65% for all eGFRcr-cys equations (Table 2 and Supplementary Table S6), particularly across mGFR 30-44, 45-59 and 60-89 mL/min. Correct classification was consistently less than 60% for eGFRcr and eGFRcys.

**Table 2:** Contingency tables showing agreement across categories of eClcr, eGFR and mGFR

	eGFRcr: CKD-EPI 2009 – Correct classification 55.5%					
mGFR (mL/min)	120+	90-119	60-89	45-59	30-44	<30
120+	57.6	42.4	0	0	0	0
90-119	12.7	70.5	16.9	0	0	0
60-89	1.4	33.1	57.3	7.6	0.6	0
45-59	0.3	6.2	44.9	38.6	9.7	0.3
30-44	0	0.9	15.2	30.8	47.3	5.8
<30	0	1.8	6.4	6.4	40.4	45
	eGFRcr: EKFC 2021 (mL/min) – Correct classification 57.6%					
mGFR (mL/min)	120+	90-119	60-89	45-59	30-44	<30
120+	38.4	61.6	0	0	0	0
90-119	6.1	62.7	31.2	0	0	0
60-89	0.6	20.5	67.4	10.6	0.9	0
45-59	0	3.4	37.2	44	15.1	0.3
30-44	0	0.4	12.1	28.6	51.3	7.6
<30	0	0.9	4.6	10.1	35.8	48.6
	eGFRcys: CKD-EPI 2012 – Correct classification 51.3%					
mGFR (mL/min)	120+	90-119	60-89	45-59	30-44	<30
120+	51	39.7	8.6	0.7	0	0
90-119	11	47.9	36.7	3.8	0.6	0
60-89	0.7	11.1	52.9	26.4	8	1
45-59	0	0.3	11.6	40.6	41.8	5.7
30-44	0	0	0.9	8.9	54.9	35.3
<30	0	0.9	0.9	1.8	13.8	82.6
	eGFRcys: EKFC 2023 (mL/min) – Correct classification 55.4%					
mGFR (mL/min)	120+	90-119	60-89	45-59	30-44	<30
120+	19.9	69.5	10.6	0	0	0
90-119	3	45.4	48.7	2.5	0.4	0
60-89	0	5.2	65.8	23.4	5.2	0.4
45-59	0	0.3	13.6	51.7	31.2	3.1
30-44	0	0	1.3	17.4	67	14.3
<30	0	0.9	0.9	1.8	31.2	65.1
	eGFRcr-cys: CKD-EPI 2012 – Correct classification 64.9%					
mGFR (mL/min)	120+	90-119	60-89	45-59	30-44	<30
120+	59.6	39.7	0.7	0	0	0
90-119	10.8	64.8	24.3	0.2	0	0
60-89	0.6	15.8	68.7	14.1	0.6	0.2
45-59	0	1.1	22.4	56.5	19	0.9
30-44	0	0.4	3.1	19.6	64.7	12.1
<30	0	0.9	1.8	1.8	22.9	72.5
	eGFRcr-cys: EKFC 2023 (mL/min) – Correct classification 62.6%					
mGFR (mL/min)	120+	90-119	60-89	45-59	30-44	<30
120+	25.8	73.5	0.7	0	0	0
90-119	4	52.7	43.2	0	0	0
60-89	0	7.6	78.5	13.6	0.1	0.2
45-59	0	0.6	24.7	58.5	15.9	0.3
30-44	0	0.4	2.7	25.4	64.3	7.1
<30	0	0.9	1.8	4.6	39.4	53.2

Values are presented as percentage (%) of individuals. Each row totals 100% to allow comparison of agreement across mGFR categories. mGFR: measured glomerular filtration rate (GFR) by single-point iohexol clearance; eClcr: estimated creatinine clearance; eGFRcr: estimated GFR by creatinine; eGFRcys: estimated GFR by cystatin C; eGFRcr-cys: estimated GFR by creatinine and cystatin C; EKFC: European Kidney Function Consortium.

### Carboplatin dosing

**Fig. 2:**
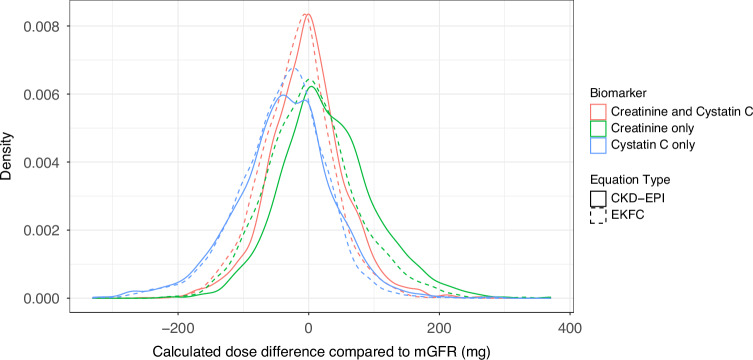
Plots of recommended dosing of carboplatin compared to mGFR. Density plots showing the difference in recommended dose of carboplatin (to achieve AUC 5 mg/mL/min) calculated by eClcr, eGFRcr, eGFRcys and eGFRcr-cys compared to mGFR. Positive values indicate a higher dose than was calculated by mGFR; negative values represent a lower dose than was calculated by mGFR. CKD-EPI: Chronic Kidney Disease Epidemiology Collaboration; EKFC: European Kidney Function Consortium; mGFR: measured glomerular filtration rate (GFR) by single-point iohexol clearance.

**Fig. 3:**
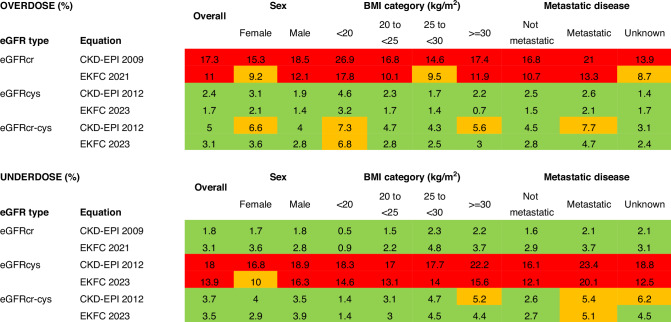
Subgroups: Proportion of individuals recommended an overdose or underdose of carboplatin compared to dose recommended by mGFR for AUC 5 mg/mL/min of carboplatin. AUC: area under the curve; BMI: body mass index; eGFRcr: estimated GFR by creatinine; eGFRcys: estimated GFR by cystatin C; eGFRcr-cys: estimated GFR by creatinine and cystatin C; EKFC: European Kidney Function Consortium; mGFR: measured glomerular filtration rate (GFR) by single-point iohexol clearance. Green: ≤5%; Amber: >5-10%; Red: >10%.

The median recommended dose of carboplatin for target AUC 5 mg/mL/min ranged from 320 mg (mGFR 30-44 mL/min) to 750 mg (mGFR 120+ mL/min). Dosing accuracy was generally best for eGFRcr-cys (Fig. 2 and Supplementary Fig. S2). Using eGFRcr would recommend a dose of carboplatin that was likely to result in “overdose” of carboplatin in around 10-20% of individuals, with similar findings across sex, BMI and metastatic disease subgroups (Fig. 3). This was 3-4 times less common when using eGFRcr-cys. Using eGFRcys, “underdose” of carboplatin was recommended in around 10-20% of individuals, with similar findings across subgroups, and was again 3-4 times less common when using eGFRcr-cys (Fig. 3).

### Subgroups: P30 and P15

There were no substantial differences in P30 or P15 when stratifying by sex (Supplementary Table S7). eGFRcr and eGFRcys equations more commonly showed inadequate accuracy (P30 < 80%) at extremes of BMI (<20 or ≥30 kg/m^2^), but accuracy of eGFRcr-cys remained adequate or optimal across all levels of BMI (Supplementary Table S8). Accuracy of all equations was lower in those with metastatic compared with not metastatic disease. eGFRcr-cys showed highest accuracy in those with metastatic disease, but P30 remained adequate or optimal for all eGFRcr-cys equations (Supplementary Table S9).

### Subgroups: bias and precision

Males showed medium or large positive bias for two eGFRcr equations, and a greater bias than was observed in females (Supplementary Table S10). Males also showed medium or large negative bias in all eGFRcys equations, while negative bias for eGFRcys was generally small in females. eGFRcr-cys equations generally showed small bias and better precision than eGFRcr or eGFRcys. Precision for all equations was better in females than in males. Across BMI subgroups, eGFRcr more often showed medium or large positive bias in both low and high BMI categories (Supplementary Table S11). eGFRcys showed small bias in those with BMI < 20 kg/m^2^, with increasing negative bias across increasing categories of BMI. eGFRcr-cys equations generally showed small bias across all BMI categories. For all equations, precision was better in lower BMI categories. Bias was similar and small for all eGFRcr-cys equations regardless of metastatic disease status, but precision was lower in those with metastatic versus not metastatic disease (Supplementary Table S12).

### Subgroups: carboplatin dosing

As for the overall group, eGFRcr-cys was less likely to recommend “overdose” than eGFRcr, and less likely to recommend “underdose” than eGFRcys, across all subgroups. It was more common for males than females, those with low BMI versus high BMI, and those with metastatic versus not metastatic disease, to be recommended “overdose” using eGFRcr (Fig. 3 and Supplementary Table S13). It was more common for males than females, those with high versus low BMI and metastatic versus not metastatic disease, to be recommended “underdose” using eGFRcys (Fig. 3 and Supplementary Table S14). eGFRcr-cys made the most consistent dosing recommendations across subgroups. Although the proportions were small, those with metastatic disease were still around twice as likely as those without metastatic disease to be recommended “overdose” or “underdose” of carboplatin using eGFRcr-cys (Fig. 3 and Supplementary Table S13 and S14)

## Discussion

In this study conducted in patients with cancer in routine care, we found that eGFRcr-cys equations provide the most accurate, least biased and most precise estimates of mGFR. eGFRcr-cys equations were more likely to classify patients correctly across a range of GFR thresholds important for treatment eligibility and provided more accurate dosing recommendations (for carboplatin) compared to eGFRcr and eGFRcys, including across subgroups by sex, BMI category and metastatic disease. The findings are in keeping with those seen in general population settings, despite concerns that the non-GFR determinants of creatinine and cystatin C may be augmented among people with cancer.

Our results are in keeping with the largest research cohort study of adults with solid organ tumours from Brazil, where the authors found that eGFRcr generally overestimated mGFR, eGFRcys underestimated mGFR, and eGFRcr-cys was most accurate[3]. Our study showed consistent findings in patients who (on average) were older, had lower BMI, lower mGFR, a different distribution of cancer types and higher burden of metastatic disease, reflecting expected differences in a routine care population compared to a prospective cohort. Nevertheless, the message appears consistent that guidance for the general population – to consider eGFRcr-cys where eGFRcr are likely to be inaccurate – can similarly be applied to populations with cancer managed in routine care.

Various participant and cancer characteristics may impact the accuracy of GFR estimates using creatinine and cystatin C. Creatinine is primarily impacted by muscle mass and function. Loss of muscle mass such as that seen in cachexia (especially in advanced cancer, liver or neurological disease) is associated with lower creatinine values and higher eGFRcr. Though the data are not definitive, cystatin C may be increased in smoking, obesity, chronic inflammation and use of medications such as thyroid hormones and glucocorticoids [5, 27], leading to lower eGFRcys. We have shown that eGFRcr and eGFRcys both have adequate (but not optimal) P30, and systematic bias (compared to mGFR in patients with cancer) in opposite directions. In a prior cohort study of 1,869 patients with cancer, wide discrepancies in eGFRcys and eGFRcr (indicating deviations in non-GFR determinants of one or both filtration markers) were associated with higher rates of supratherapeutic vancomycin trough level (>30 μg/mL), trimethoprim-sulfamethoxazole-related hyperkalemia (>5.5 mEq/L), baclofen toxic effects, supratherapeutic digoxin levels (>2.0 ng/mL), and a higher likelihood of increased mortality at 30 days [28].

The American Society of Onco-Nephrology recommends use eGFR equations incorporating both creatinine and cystatin C in patients with cancer, with use of mGFR for patients with borderline eligibility for treatments or clinical trials [14]. The potential benefits of testing cystatin C more widely in patients with cancer is two-fold. First, the use of both filtration markers as eGFRcr-cys partly attenuates the inaccuracy due to deviation in non-GFR determinants of each marker and provides a more accurate estimate than either marker alone, plausibly improving the accuracy of treatment selection and dosing, and reducing the likelihood of systemic adverse events [28]. Second, this would highlight individuals in whom the difference between eGFRcr and eGFRcys is large, and where treatment selection or dose would be different for eGFRcr and eGFRcys. This should prompt consideration of potential sources of error (i.e., deviations in the non-GFR determinants of both creatinine and cystatin C) and consideration of whether mGFR – requiring additional time, cost and expertise – is warranted [4]. We are not aware of any study that has shown that improved accuracy of drug dosing, using an equation that better estimates mGFR, has directly led to an improvement in clinical outcomes, neither in cancer populations nor in general population settings. The implications of using more accurate estimates of GFR, such as eGFRcr-cys, for cancer treatment safety and clinical outcomes would benefit from prospective confirmation, which would require routine testing of cystatin C in clinical cancer trials.

In this study, we assessed the performance of 13 equations to estimate GFR using creatinine and cystatin C relative to mGFR. Though there appeared to be greater variation in performance between filtration markers than between equations, some additional variation existed also between equations. Most oncology trials have used the Cockcroft-Gault (CG) [29] equation to guide participant inclusion and drug dosing [26]; however, CG estimates creatinine clearance (eClcr) rather than GFR, many limitations of the CG equation are recognised, and this equation is no longer recommended for use [30]. Current European Renal Association [31] and European Federation of Clinical Chemistry and Laboratory Medicine [32] policies recommend eGFRcr using CKD-EPI 2009 (without applying a race correction factor). As reported before in the SCREAM cohort [33], eGFRcr by CKD-EPI 2021 tended to over-estimate mGFR more than other eGFRcr equations, with potential for overexposure to treatments with narrow therapeutic index. Like the general population, there is probably not a single best equation that can be recommended for patients with cancer globally and some regional-specific calibration of clinical guidance may be required. However, data from this and other studies suggest that eGFRcr-cys is more accurate than either eGFRcr or eGFRcys.

### Strengths and limitations

Our study is likely to be the largest routine care cohort worldwide with information on mGFR, creatinine, and cystatin C using state-of-the-art measures, and with cancer registry linkage, to facilitate this type of analysis. However, we acknowledge some limitations. First, included patients were those who had been referred for mGFR testing in routine care. This indication bias is likely to have selected a population that is not representative of all patients with cancer. For example: i) patients in whom filtration markers (either creatinine, cystatin C or both) were thought to be insufficiently accurate, or those who were more likely to have large discrepancies between eGFRcr and eGFRcys; ii) patients who were under the care of clinicians who were more conscious of the importance of kidney function in cancer management; iii) patients under the care of clinicians managing specific cancer types or treatments where measuring GFR is more strongly advocated. Second, we have compared the performance of eGFR against mGFR by iohexol clearance, but the latter is also subject to variability depending on the exogenous marker, analytical methods, and sample collection protocol [34, 35]. mGFR data in this study were conducted within a single centre, with consistency in measurement, and were subject to internal and external validation, which minimised analytical variability. Nevertheless, biological variability of up to 10% may still be expected within an individual [36], which will have had some impact on agreement between the measures, particularly across fixed eligibility and dosing thresholds. Third, SCREAM does not contain information about prescription or delivery of cancer treatments. In this study, it was not possible to assess the actual treatments received according to kidney function or their association with outcomes. Fourth, we included patients with any solid organ or haematological malignancy and thus there was substantial heterogeneity in the group with respect to cancer type and stage. We cannot be certain that our findings apply equally to all people diagnosed with cancer or at all stages of disease. Fifth, though accurate data on race are not available, the SCREAM cohort is a predominantly Caucasian population living in Sweden, and we cannot guarantee generalisability to other populations or geographical regions [37].

## Conclusion

eGFRcr-cys equations provide the most accurate estimates of mGFR in patients with cancer. The incorporation of cystatin C with creatinine for GFR estimation would be relatively inexpensive and practically simple, with the potential to improve dosing and classification across treatment thresholds compared to creatinine alone.

## Supplementary information

Supplementary data file

## Data Availability

Analysis code and metadata will be available on publication at the project GitHub page: https://github.com/jennifer-s-lees/scream_cancer_egfr_accuracy_public. Individual participant-level data are available on application from the SCREAM resource: https://ki.se/en/research/research-areas-centres-and-networks/research-groups/cardio-renal-epidemiology-juan-jesus-carreros-research-group#tab-scream

## Code availability

Analysis code and metadata will be available on publication at the project GitHub page: https://github.com/jennifer-s-lees/scream_cancer_egfr_accuracy_public.
