# Supplementary material for: Accuracy of glomerular filtration rate estimates among patients with cancer
Source: Br J Cancer. 2025 Sep 19;133(11):1652–9. doi: 10.1038/s41416-025-03190-3 (PMC12644916; doi:10.1038/s41416-025-03190-3)
Supplement: Supplementary file 1 — Supplementary data file [file 41416_2025_3190_MOESM1_ESM.pdf]

Supplementary data file

"Accuracy of glomerular filtration rate estimates among patients with cancer"

Supplementary methods 2

Selection of GFR estimating equations 2

Supplementary Tables 5

Table S1: ICD-10 codes for cancer diagnosis and type 5

Table S2: Baseline data comparing those with missing height or weight to those with available height and weight 6

Table S3: ICD-10 codes for comorbidities 7

Table S3: Baseline data: additional data and stratified by sex 8

Table S4: P30, P15 and Bias for remaining equations not reported in main paper 10

Table S5: Sex subgroup - Accuracy 15

Table S6: Sex subgroup - Bias and Precision 17

Table S7: Bias for eGFR and mGFR values indexed to body surface area 11

Table S8: Contingency tables showing agreement across categories of eGFR and mGFR 12

Table S9: Proportion of individuals recommended an overdose or underdose of carboplatin compared to dose recommended by mGFR for AUC 5mg/mL/min of carboplatin 19

Table S10: BMI subgroups - Accuracy 16

Table S11: BMI subgroups - Bias and Precision 20

Table S12: Metastatic disease subgroups - Accuracy 17

Table S13: Metastatic disease subgroups - Bias and Precision 21

Table S14: Subgroups: Proportion of individuals recommended an overdose of carboplatin compared to dose recommended by mGFR for AUC 5mg/mL/min of carboplatin 22

Table S14: Subgroups: Proportion of individuals recommended an underdose of carboplatin compared to dose recommended by mGFR for AUC 5mg/mL/min of carboplatin 23

Supplementary Figures 24

Figure S1: Bland-Altman plots for agreement between mGFR and eGFR 24

Figure S2: Subgroups: Plots of recommended dosing of carboplatin compared to mGFR 25

Supplementary methods

Selection of GFR estimating equations

In the main paper, we present estimated glomerular filtration rate (eGFR) based on serum or plasma values of creatinine (eGFRcr), cystatin C (eGFRcys) or the combination of both filtration markers (eGFRcr-cys), calculated by the Chronic Kidney Disease Epidemiology Collaboration (CKD-EPI 2009/2012) and European Kidney Function Consortium (EKFC 2021/2023) equations. The CKD-EPI 2009/2012 equations (without applying a race correction factor for creatinine-based equations) are currently recommended by the European Renal Association[1] and European Federation of Clinical Chemistry and Laboratory Medicine[2] policies. The EKFC equations have been developed and extensively validated in Europe and are supported as suitable alternative equations for European populations[3]. eGFRcr, eGFRcys and eGFRcr-cys exist in various other forms (with and without race coefficients). Specific eGFRcr equations have also been developed to optimise performance in patients with cancer (CamGFRv2[4]) and reportedly perform better than equations developed for the general population, but perform less well in the general population[5] and there is concern about the practicality of using of disease-specific or treatment-specific eGFR equations[6]. Most oncology trials have used the Cockcroft-Gault (CG) equation to guide inclusion and dosing[7]; however, CG is supposed to estimate creatinine clearance (eClcr; in mL/min) rather than eGFR. Moreover, many limitations of the CG equation are recognised, and this equation is no longer recommended for use in international guidance[8]; however, we include eClcr to illustrate performance compared with other eGFR equations.

The additional equations we have included are as follows:

- eClcr: *CG[7]
- eGFRcr: ^CKD-EPI 2021[9]; ^Lund-Malmö Revised (LMR)[10]; *CamGFRv2[4]
- eGFRcys: ^Caucasian, Asian, pediatric and adult (CAPA)[11]
- eGFRcr-cys: ^CKD-EPI 2021[9]; ^LMR-CAPA (the mean of LMR and CAPA)

* CG and CamGFRv2 equations are reported in mL/min.

^Indexed eGFR values (in mL/min/1.73m^2) were converted to non-indexed eGFR (as for equations included in main paper) and reported in mL/min to ensure comparability between eGFR and mGFR and to optimise accuracy for drug dosing calculations.

Supplementary References:

1. Gansevoort RT, Anders H-J, Cozzolino M et al. What should European nephrology do with the new CKD-EPI equation? Nephrology Dialysis Transplantation 2023; 38: 1–6. DOI:10.1093/ndt/gfac254
2. Delanaye P, Schaeffner E, Cozzolino M et al. The new, race-free, Chronic Kidney Disease Epidemiology Consortium (CKD-EPI) equation to estimate glomerular filtration rate: is it applicable in Europe? A position statement by the European Federation of Clinical Chemistry and Laboratory Medicine (EFLM). Clinical chemistry and laboratory medicine 2023; 61: 44–47. DOI:10.1515/cclm-2022-0928
3. Cavalier E, Zima T, Datta P et al. Recommendations for European laboratories based on the KDIGO 2024 Clinical Practice Guideline for the Evaluation and Management of Chronic Kidney Disease. Clinical Chemistry and Laboratory Medicine (CCLM) 2025; 63: 525–534. DOI:10.1515/cclm-2024-1082
4. Williams EH, Flint TR, Connell CM et al. CamGFR v2: A new model for estimating the glomerular filtration rate from standardized or non-standardized creatinine in patients with cancer. Clinical Cancer Research 2021; 27: 1381–1390. DOI:10.1158/1078-0432.CCR-20-3201
5. Couture SJ, Tighiouart H, Costa e Silva VT, Inker LA, Levey AS. Evaluation of the Cambridge GFR Estimating Equation in a Diverse Population. Journal of the American Society of Nephrology 2021; 32: 712–712. DOI:10.1681/ASN.20213210S1712c
6. Inker LA, Chami A, Levey AS. Do We Need a New Creatinine-Based Estimated GFR Equation for Kidney Transplant Recipients? American Journal of Kidney Diseases 2024; 83: 257–259. DOI:10.1053/j.ajkd.2023.08.020
7. Cockcroft DW, Gault MH. Prediction of creatinine clearance from serum creatinine. Nephron 1976; 16: 31–41. DOI:10.1159/000180580
8. St. Peter WL, Bzowyckyj AS, Anderson-Haag T et al. Moving forward from Cockcroft-Gault creatinine clearance to race-free estimated glomerular filtration rate to improve medication-related decision-making in adults across healthcare settings: A consensus of the National Kidney Foundation Workgroup for Implementation of Race-Free eGFR-Based Medication-Related Decisions. American Journal of Health-System Pharmacy 2024;DOI:10.1093/ajhp/zxae317
9. Inker L, Eneanya N, Coresh J et al. New Creatinine- and Cystatin C–Based Equations to Estimate GFR without Race. New Engl J Med 2021;DOI:10.1056/NEJMoa2102953
10. Björk J, Grubb A, Sterner G, Nyman U. Revised equations for estimating glomerular filtration rate based on the Lund-Malmö Study cohort. Scandinavian Journal of Clinical

and Laboratory Investigation *[Internet]* 2011; [cited 2025 Jan 22] 71: 232–239. DOI:10.3109/00365513.2011.557086

11. Grubb A, Horio M, Hansson L-O et al. Generation of a New Cystatin C–Based Estimating Equation for Glomerular Filtration Rate by Use of 7 Assays Standardized to the International Calibrator. Clinical Chemistry 2014; 60: 974–986. DOI:10.1373/clinchem.2013.220707

Supplementary Tables

Table S1: ICD-10 codes for cancer diagnosis and type

Cancer type	ICD-10 code
Lip, oral and pharynx	C00-14
Upper GI	C15-17
Colorectal	C18-20
Anal	C21
Abdominal organs	C22-26
Head and neck	C30-32
Lung and bronchus	C33-34
Mediastinal	C37-39
Bone	C40-41
Melanoma	C43
Soft tissue	C45-49
Breast	C50
Female genital organs	C51-58
Prostate	C61
Male genital organs	C60, C62-63
Kidney	C64-65
Ureter	C66
Bladder	C67
Other urinary tract	C68
Central nervous system	C69-72
Endocrine	C73-75, C7A, C7B
Other	C76-80, C96
Lymphoma	C81-86, C88
Leukaemia	C91-96
Myeloma	C90

ICD-10: International Classification of Disease Version 10.

Table S2: Baseline data comparing those with missing height or weight to those with available height and weight

	Missing height or weight	Height and weight available	p
**N**	517	1837	
**Age (median [IQR]) years**	64.0 [47.0, 72.0]	68.0 [61.0, 74.0]	<0.001
**Female: n(%)**	146 (28.2)	707 (38.5)	<0.001
**Cancer type (%)**			<0.001
**Bladder**	177 (34.2)	599 (32.6)	
**Colorectal**	34 (6.6)	229 (12.5)	
**Female genital organs**	19 (3.7)	129 (7.0)	
**Kidney/ureter**	27 (5.2)	120 (6.5)	
**Lung or bronchus**	19 (3.7)	428 (23.3)	
**Male genital organs**	154 (29.8)	72 (3.9)	
**Other**	87 (16.8)	260 (14.2)	
**Metastatic (%)**			0.013
**Not metastatic**	376 (72.7)	1212 (66.0)	
**Metastatic**	68 (13.2)	319 (17.4)	
**Unknown**	73 (14.1)	306 (16.7)	
**Diabetes mellitus: n(%)**	65 (12.6)	379 (20.6)	<0.001
**Hypertension: n(%)**	237 (45.8)	950 (51.7)	0.021
**Cardiovascular disease: n(%)**	131 (25.3)	576 (31.4)	0.01
**Liver disease: n(%)**	22 (4.3)	232 (12.6)	<0.001
**mGFR (mean (SD)) mL/min/1.73m^2**	70.6 (26.3)	68.2 (24.8)	0.055

mGFR: measured glomerular filtration rate (GFR) by single-point iohexol clearance. Indexed value (in mL/min/1.73m^2) displayed due to lack of height and weight data to de-index for body surface area.

Table S3: ICD-10 codes for comorbid conditions

**Comorbidity**	**ICD-10 codes**
Diabetes mellitus	E10-E14
Hypertension	I10-15
Cardiovascular disease	Acute coronary syndrome and/or other ischaemic heart disease and/or heart failure and/or stroke and/or other cerebrovascular disease and/or arrhythmia including atrial fibrillation and/or peripheral vascular disease.
Components of cardiovascular disease	
Acute coronary syndrome	I200, I21-22
Other ischaemic heart disease	I201, I208, I209, I24-25
Heart failure	I110, I130, I132, I50
Stroke	I60-64, I693, I694, I698
Other cerebrovascular disease	I65-I69, G450-453, G458-459, G46
Arrhythmia including atrial fibrillation	I44-49
Peripheral vascular disease	I70, I72-73
Liver disease	B18, I982, K70-77

ICD-10: International Classification of Disease Version 10.

Table S3: Baseline data: additional data and stratified by sex

	Overall	Male	Female	p
**N (%)**	1837	1130	707	
**Age (years)**	68 [61, 74]	68 [61, 73]	68 [61, 74]	0.04
**Body mass index: kg/m^2**	25.3 (4.5)	25.6 (4.1)	24.8 (5.0)	<0.001
**Body surface area: m^2**	1.9 (0.2)	2.0 (0.2)	1.7 (0.2)	<0.001
**Comorbidity count**	1.0 [0.0, 2.0]	1.0 [0.0, 3.0]	1.0 [0.0, 2.0]	0.003
**Cancer type: n(%)**				<0.001
**Bladder**	599 (32.6)	439 (38.8)	160 (22.6)	
**Colorectal**	229 (12.5)	183 (16.2)	46 (6.5)	
**Female genital organs**	129 (7.0)	0 (0.0)	129 (18.2)	
**Kidney/ureter**	120 (6.5)	84 (7.4)	36 (5.1)	
**Lung and bronchus**	428 (23.3)	212 (18.8)	216 (30.6)	
**Male genital organs**	72 (3.9)	72 (6.4)	0 (0.0)	
**Other**	260 (14.2)	140 (12.4)	120 (17.0)	
**Metastatic: n(%)**				<0.001
**Not metastatic**	1212 (66.0)	871 (77.1)	341 (48.2)	
**Metastatic**	319 (17.4)	172 (15.2)	147 (20.8)	
**Unknown**	306 (16.7)	87 (7.7)	219 (31.0)	
**Systemic glucocorticoid use: n(%)**	621 (33.8)	318 (28.1)	303 (42.9)	<0.001
**Diabetes: n(%)**	379 (20.6)	272 (24.1)	107 (15.1)	<0.001
**Hypertension: n(%)**	950 (51.7)	573 (50.7)	377 (53.3)	0.297
**Cardiovascular disease: n(%)**	576 (31.4)	378 (33.5)	198 (28.0)	0.017
**Liver disease: n(%)**	232 (12.6)	171 (15.1)	61 (8.6)	<0.001
**Values non-indexed to body surface area (mL/min)**				
**mGFR**	74.6 (29.6)	80.8 (30.6)	64.8 (25.0)	<0.001
**eClcr Cockcroft and Gault**	75.7 (31.6)	80.6 (31.6)	68.0 (30.1)	<0.001
**eGFRcr CamGFRv2**	71.5 (23.4)	77.4 (23.3)	62.0 (20.2)	<0.001
**eGFRcr CKD-EPI 2009**	80.6 (28.3)	86.7 (28.2)	70.9 (25.6)	<0.001
**eGFRcr CKD-EPI 2021**	85.2 (28.8)	91.7 (28.6)	74.8 (26.0)	<0.001
**eGFRcr EKFC 2021**	75.6 (26.3)	81.6 (26.1)	66.2 (23.7)	<0.001
**eGFRcr LMR**	73.1 (25.2)	77.7 (25.0)	65.8 (23.8)	<0.001
**eGFRcys CKD-EPI 2012**	67.4 (30.5)	72.6 (31.6)	58.9 (26.5)	<0.001

**eGFRcys EKFC 2023**	66.7 (25.3)	70.5 (26.1)	60.6 (22.6)	<0.001
**eGFRcys CAPA**	66.3 (28.0)	69.6 (28.4)	61.1 (26.7)	<0.001
**eGFRcr-cys CKD-EPI 2012**	74.1 (29.1)	79.6 (29.5)	65.3 (26.3)	<0.001
**eGFRcr-cys CKD-EPI 2021**	76.8 (30.1)	82.6 (30.4)	67.7 (27.3)	<0.001
**eGFRcr-cys EKFC 2023**	71.1 (24.5)	76.0 (24.8)	63.4 (22.1)	<0.001
**eGFRcr-cys LMR-CAPA**	69.7 (25.0)	73.6 (25.0)	63.4 (23.7)	<0.001
**Values indexed to body surface area (mL/min/1.73m^2)**				
**mGFR**	68.2 (24.8)	70.1 (25.0)	65.2 (24.2)	<0.001
**eGFRcr CKD-EPI 2009**	74.0 (24.1)	75.6 (23.6)	71.4 (24.8)	<0.001
**eGFRcr CKD-EPI 2021**	78.2 (24.4)	80.0 (23.8)	75.3 (25.1)	<0.001
**eGFRcr EKFC 2021**	69.3 (22.2)	71.1 (21.6)	66.5 (22.9)	<0.001
**eGFRcr LMR**	67.1 (21.8)	67.8 (20.9)	66.2 (23.1)	0.128
**eGFRcys CKD-EPI 2012**	61.7 (26.4)	63.2 (26.5)	59.2 (26.0)	0.002
**eGFRcys EKFC 2023**	61.1 (21.7)	61.3 (21.6)	60.9 (21.9)	0.705
**eGFRcys CAPA**	60.8 (24.8)	60.5 (23.8)	61.4 (26.3)	0.45
**eGFRcr-cys CKD-EPI 2012**	67.9 (25.1)	69.3 (24.6)	65.7 (25.8)	0.002
**eGFRcr-cys CKD-EPI 2021**	70.4 (26.0)	71.9 (25.4)	68.1 (26.7)	0.003
**eGFRcr-cys EKFC 2023**	65.2 (20.7)	66.2 (20.4)	63.7 (21.2)	0.013
**eGFRcr-cys LMR-CAPA**	64.0 (21.7)	64.1 (20.8)	63.8 (23.1)	0.74

Mean (standard deviation; SD) or median (interquartile range; IQR) unless otherwise specified. eClcr: estimated creatinine clearance; eGFRcr: estimated GFR by creatinine; eGFRcys: estimated GFR by cystatin C; eGFRcr-cys: estimated GFR by creatinine and cystatin C; CAPA: Caucasian, Asian, pediatric and adult; CKD-EPI: Chronic Kidney Disease Epidemiology Collaboration; EKFC: European Kidney Function Consortium; LMR: Lund-Malmö Revised; mGFR: measured glomerular filtration rate (GFR) by single-point plasma clearance of iohexol;

Table S4: P30, P15 and Bias for remaining equations not reported in main paper

		Accuracy		Bias	Precision
eGFR type	eGFR	P30 (%)	P15 (%)	Median difference (mL/min)	IQR of the difference (mL/min)
eClcr	CG	83.7	51.9	-1	19.4
eGFRcr	CamGFRv2	86.9	54	-2.7	18.8
	CKD-EPI 2021	73.7	46.8	9.7	19
	LMR	86.8	54.9	-1.3	18.3
eGFRcys	CAPA	81.8	48.5	-7.4	18.5
eGFRcr-cys	CKD-EPI 2021	90.3	64.3	1.9	14.8
	LMR CAPA	92.3	62.3	-4.1	14.8

Percentage of patients with estimated values that were within 30% of mGFR (P30) and within 15% of mGFR (P15). Bias was estimated as median difference of eClcr or eGFR minus mGFR. IQR: interquartile range of the bias. CAPA: Caucasian, Asian, pediatric and adult; CKD-EPI: Chronic Kidney Disease Epidemiology Collaboration; eGFRcr: estimated GFR by creatinine; eGFRcys: estimated GFR by cystatin C; eGFRcr-cys: estimated GFR by creatinine and cystatin C; LMR: Lund-Malmö Revised; mGFR: measured glomerular filtration rate (GFR) by single-point iohexol clearance. Green: optimal accuracy/small bias; Amber: adequate accuracy/medium bias; Red: inadequate accuracy/large bias.

Table S5: Bias for eGFR and mGFR values indexed to body surface area

eGFR	Bias (mL/min/1.73m^2)	IQR (mL/min/1.73m^2)
eGFRcr: CKD-EPI 2009	4.8	17.6
eGFRcr: CKD-EPI 2021	8.9	17.9
eGFRcr: EKFC 2021	0.6	16.4
eGFRcr: LMR	-1.3	17.1
eGFRcys: CKD-EPI 2012	-6.5	16.6
eGFRcys: EKFC 2023	-6.1	15.9
eGFRcys: CAPA	-6.9	16.8
eGFRcr-cys: CKD-EPI 2012	-0.5	13.3
eGFRcr-cys: CKD-EPI 2021	1.8	13.9
eGFRcr-cys: EKFC 2023	-2.7	13.6
eGFRcr-cys: LMR CAPA	-3.8	13.5

Results presented for equations that do not internally correct for body surface area. Bias was estimated as median difference of eGFR minus mGFR. IQR: interquartile range of the bias. CAPA: Caucasian, Asian, pediatric and adult; CKD-EPI: Chronic Kidney Disease Epidemiology Collaboration; estimated GFR by serum creatinine; eGFRcys: estimated GFR by serum cystatin C; eGFRcr-cys: estimated GFR by serum creatinine and cystatin C; EKFC: European Kidney Function Consortium; LMR: Lund-Malmö Revised; mGFR: measured glomerular filtration rate (GFR) by single-point iohexol clearance. Green: small bias; Amber: medium bias; Red: large bias.

Table S6: Contingency tables showing agreement across categories of eGFR and mGFR

	eClcr: CG (mL/min) – Correct classification 53.5%					
mGFR (mL/min)	120+	mGFR (mL/min)	120+	mGFR (mL/min)	120+	mGFR (mL/min)
120+	56.3	120+	56.3	120+	56.3	120+
90-119	19	90-119	19	90-119	19	90-119
60-89	2.7	60-89	2.7	60-89	2.7	60-89
45-59	0.3	45-59	0.3	45-59	0.3	45-59
30-44	0	30-44	0	30-44	0	30-44
<30	0	<30	0	<30	0	<30
	eGFRcr: CamGFRv2 – Correct classification 56.7%					
mGFR (mL/min)	120+	90-119	60-89	45-59	30-44	<30
120+	18.5	76.2	5.3	0	0	0
90-119	1.7	44.1	54	0.2	0	0
60-89	0.2	10.1	76.5	12.4	0.6	0.1
45-59	0	1.1	36.1	50	12.5	0.3
30-44	0	0.4	9.8	31.7	50.9	7.1
<30	0	0.9	4.6	7.3	35.8	51.4
	eGFRcr: CKD-EPI 2021 – Correct classification 50.9%					
mGFR (mL/min)	120+	90-119	60-89	45-59	30-44	<30
120+	73.5	26.5	0	0	0	0
90-119	18.8	72.8	8.4	0	0	0
60-89	2.1	46.1	47.4	4	0.4	0

45-59	0.3	10.8	51.4	32.1	5.4	0
30-44	0.4	1.8	20.1	36.2	38.4	3.1
<30	0	2.8	5.5	11.9	42.2	37.6
	eGFRcr: LMR – Correct classification 57.0%					
mGFR (mL/min)	120+	90-119	60-89	45-59	30-44	<30
120+	25.8	71.5	2.6	0	0	0
90-119	4.2	53	42.8	0	0	0
60-89	0.6	14.6	71.8	12.1	0.7	0.2
45-59	0	2.3	35.8	47.7	13.9	0.3
30-44	0	0.4	10.7	27.2	48.2	13.4
<30	0	0.9	5.5	7.3	30.3	56
	eGFRcys: CAPA – Correct classification 49.8%					
mGFR (mL/min)	120+	90-119	60-89	45-59	30-44	<30
120+	31.1	55	13.2	0.7	0	0
90-119	9.3	40.1	46.2	3.8	0.6	0
60-89	1	8.6	55.3	26.9	7.6	0.6
45-59	0	0.3	12.8	45.2	37.2	4.5
30-44	0	0	1.3	13.4	58	27.2
<30	0	0.9	0.9	1.8	22	74.3
	eGFRcr-cys: CKD-EPI 2021 – Correct classification 63.7%					
mGFR (mL/min)	120+	90-119	60-89	45-59	30-44	<30
120+	72.2	27.2	0.7	0	0	0
90-119	14.8	66.7	18.4	0.2	0	0

60-89	1	22.8	64.9	10.6	0.5	0.2
45-59	0	1.4	28.7	53.7	15.3	0.9
30-44	0	0.4	4	24.6	60.7	10.3
<30	0	0.9	1.8	1.8	26.6	68.8
	eGFRcr-cys: LMR CAPA – Correct classification 61.7%					
mGFR (mL/min)	120+	90-119	60-89	45-59	30-44	<30
120+	24.5	70.9	4.6	0	0	0
90-119	2.7	50.4	46.8	0	0	0
60-89	0.5	6.8	74.3	17.7	0.5	0.2
45-59	0	0.6	18.8	61.9	18.2	0.6
30-44	0	0	2.7	22.3	63.8	11.2
<30	0	0.9	1.8	2.8	29.4	65.1

Values are presented as percentage (%) of individuals. Each row totals 100% to allow comparison of agreement across mGFR categories. CAPA: Caucasian, Asian, pediatric and adult; CKD-EPI: Chronic Kidney Disease Epidemiology Collaboration; estimated GFR by serum creatinine; eGFRcys: estimated GFR by serum cystatin C; eGFRcr-cys: estimated GFR by serum creatinine and cystatin C; LMR: Lund-Malmö Revised; mGFR: measured glomerular filtration rate (GFR) by single-point iohexol clearance.

Table S7: Sex subgroup - Accuracy

	**Female**		**Male**	
eGFR	**P30 (%)**	**P15 (%)**	**P30 (%)**	**P15 (%)**
eClcr: Cockcroft and Gault	81	51.2	85.4	52.4
eGFRcr: CamGFRv2	86.1	53.7	87.5	54.1
eGFRcr: CKD-EPI 2009	81.8	51.8	80.8	53
eGFRcr: CKD-EPI 2021	74.3	45.5	73.3	47.7
eGFRcr: EKFC 2021	87	57.1	85.1	57.4
eGFRcr: LMR	86.5	58.1	86.9	52.9
eGFRcys: CKD-EPI 2012	82.5	48.4	81.6	48.8
eGFRcys: EKFC 2023	87.1	57.1	84.7	50.9
eGFRcys: CAPA	83.2	52.1	80.9	46.2
eGFRcr-cys: CKD-EPI 2012	90.6	63.6	93	67.4
eGFRcr-cys: CKD-EPI 2021	88.6	60.7	91.4	66.6
eGFRcr-cys: EKFC 2023	92.3	65.9	93.6	65.8
eGFRcr-cys: LMR CAPA	91.5	65.9	92.8	60

Percentage of patients with estimated values that were within 30% of mGFR (P30) and within 15% of mGFR (P15). CAPA: Caucasian, Asian, pediatric and adult; CKD-EPI: Chronic Kidney Disease Epidemiology Collaboration; eClcr: estimated creatinine clearance; eGFRcr: estimated GFR by creatinine; eGFRcys: estimated GFR by cystatin C; eGFRcr-cys: estimated GFR by creatinine and cystatin C; EKFC: European Kidney Function Consortium; LMR: Lund-Malmö Revised; mGFR: measured glomerular filtration rate (GFR) by single-point iohexol clearance. Green: optimal accuracy; Amber: adequate accuracy; Red: inadequate accuracy.

Table S8: BMI subgroups - Accuracy

	P30				P15			
**eGFR**	<20	20 to <25	25 to <30	≥30	<20	20 to <25	25 to <30	≥30
eClcr: Cockcroft and Gault	81.7	83.8	86.4	79.8	54.8	51.9	52.6	52.7
eGFRcr: CamGFRv2	82.3	88.7	90	81	63.4	56.8	51.9	51.9
eGFRcr: CKD-EPI 2009	69.9	81.4	84.7	79.1	40.3	55.1	52.4	52.7
eGFRcr: CKD-EPI 2021	62.9	73	76.5	73.6	29.6	47.1	50.4	48.1
eGFRcr: EKFC 2021	78	86.7	88.4	83.3	58.1	59	57.2	53.1
eGFRcr: LMR	78.5	88	88.6	84.5	56.5	58.5	52.4	50
eGFRcys: CKD-EPI 2012	79.6	84.7	84.2	76	46.2	48.5	50.9	49.6
eGFRcys: EKFC 2023	83.9	87.6	87.1	83.7	53.2	55.7	53.2	50.8
eGFRcys: CAPA	79.6	85.3	83.4	77.9	45.7	51.5	49.1	46.1
eGFRcr-cys: CKD-EPI 2012	90.3	93.8	94	90.3	65.1	67.2	67.5	61.6
eGFRcr-cys: CKD-EPI 2021	87.6	91.4	92.5	89.9	59.1	66.2	65	62.4
eGFRcr-cys: EKFC 2023	89.8	94.4	94.9	90.7	71	68.9	65.8	59.3
eGFRcr-cys: LMR CAPA	91.9	95.1	93.2	89.1	70.4	66.8	59.9	58.1

Percentage of patients with estimated values that were within 30% of mGFR (P30) and within 15% of mGFR (P15). CAPA: Caucasian, Asian, pediatric and adult; CKD-EPI: Chronic Kidney Disease Epidemiology Collaboration; eClcr: estimated creatinine clearance; eGFRcr: estimated GFR by creatinine; eGFRcys: estimated GFR by cystatin C; eGFRcr-cys: estimated GFR by creatinine and cystatin C; EKFC: European Kidney Function Consortium; LMR: Lund-Malmö Revised; mGFR: measured glomerular filtration rate (GFR) by single-point iohexol clearance. Green: optimal accuracy; Amber: adequate accuracy; Red: inadequate accuracy.

Table S9: Metastatic disease subgroups - Accuracy

	P30			P15		
**eGFR**	Not metastatic	Metastatic	Unknown	Not metastatic	Metastatic	Unknown
eClcr: Cockcroft and Gault	85.5	80.6	81	54.5	48	49.3
eGFRcr: CamGFRv2	88.9	85.6	83.7	56.1	51.1	55.9
eGFRcr: CKD-EPI 2009	83.2	75.2	78.4	54.4	45.1	52
eGFRcr: CKD-EPI 2021	75	66.8	72.9	49	39.8	43.8
eGFRcr: EKFC 2021	87.6	82.1	83	58.4	53	58.5
eGFRcr: LMR	88.3	83.1	84.3	55.9	47.3	59.8
eGFRcys: CKD-EPI 2012	84.4	77.1	82.4	50.7	45.8	46.7
eGFRcys: EKFC 2023	88.8	81.2	83	55.5	49.5	52.3
eGFRcys: CAPA	85	75.9	83	51.2	42.6	49.3
eGFRcr-cys: CKD-EPI 2012	94.4	89.7	91.2	68.2	59.6	66
eGFRcr-cys: CKD-EPI 2021	92.9	86.8	88.9	66.5	58.9	62.7
eGFRcr-cys: EKFC 2023	95.2	90.3	90.5	67.7	62.4	67.3
eGFRcr-cys: LMR CAPA	95.3	88.7	90.2	64.3	58.6	66.7

Percentage of patients with estimated values that were within 30% of mGFR (P30) and within 15% of mGFR (P15). CAPA: Caucasian, Asian, pediatric and adult; CKD-EPI: Chronic Kidney Disease Epidemiology Collaboration; eClcr: estimated creatinine clearance; eGFRcr: estimated GFR by creatinine; eGFRcys: estimated GFR by cystatin C; eGFRcr-cys: estimated GFR by creatinine and cystatin C; EKFC: European Kidney Function Consortium; LMR: Lund-Malmö Revised; mGFR: measured glomerular filtration rate (GFR) by single-point iohexol clearance. Green: optimal accuracy; Amber: adequate accuracy; Red: inadequate accuracy.

Table S10: Sex subgroup - Bias and Precision

	**Female**		**Male**	
	**Bias**	**Precision**	**Bias**	**Precision**
**eGFR**	Median difference (mL/min)	IQR of the difference (mL/min)	Median difference (mL/min)	IQR of the difference (mL/min)
eClcr: Cockcroft and Gault	0.5	17.8	-1.9	20.1
eGFRcr: CamGFRv2	-2.8	16.8	-2.7	20.8
eGFRcr: CKD-EPI 2009	4.8	16.5	5.3	20.5
eGFRcr: CKD-EPI 2021	8.6	16.8	10.4	20.9
eGFRcr: EKFC 2021	0.7	15.2	0.7	19.5
eGFRcr: LMR	0.3	15.5	-2.7	20.5
eGFRcys: CKD-EPI 2012	-5.5	16.2	-7.9	19.3
eGFRcys: EKFC 2023	-3.1	15.7	-9.2	18
eGFRcys: CAPA	-3.7	16.7	-10.2	19.1
eGFRcr-cys: CKD-EPI 2012	-0.1	13.5	-0.8	15.4
eGFRcr-cys: CKD-EPI 2021	2.3	14.5	1.5	15.8
eGFRcr-cys: EKFC 2023	-1.4	12.9	-3.9	15.6
eGFRcr-cys: LMR CAPA	-1.7	12	-6.1	15.6

Bias was estimated as median difference of eClcr or eGFR minus mGFR. IQR: interquartile range of the bias. CAPA: Caucasian, Asian, pediatric and adult; CKD-EPI: Chronic Kidney Disease Epidemiology Collaboration; eClcr: estimated creatinine clearance; eGFRcr: estimated GFR by creatinine; eGFRcys: estimated GFR by cystatin C; eGFRcr-cys: estimated GFR by creatinine and cystatin C; EKFC: European Kidney Function

Consortium; LMR: Lund-Malmö Revised; mGFR: measured glomerular filtration rate (GFR) by single-point iohexol clearance. Green: small bias; Amber: medium bias; Red: large bias.

Table S11: BMI subgroups - Bias and Precision

	Bias				Precision			
	Median difference (mL/min)				IQR of the difference (mL/min)			
**eGFR**	<20	20 to <25	25 to <30	≥30	<20	20 to <25	25 to <30	≥30
eClcr: Cockcroft and Gault	0.6	-0.2	-1.8	1	15.9	18.5	20.2	22.7
eGFRcr: CamGFRv2	0.4	-2.9	-3.6	-0.5	12.6	17.5	19.7	20.9
eGFRcr: CKD-EPI 2009	11	5.2	3.8	5.7	14.6	17.1	20.1	21.1
eGFRcr: CKD-EPI 2021	14.9	9.7	8.6	10.6	16.1	17.9	20.9	21.9
eGFRcr: EKFC 2021	5.1	1	-1.1	0.9	13.5	16.3	18.6	20.8
eGFRcr: LMR	4.3	-1.2	-3.6	-1.8	12.8	16.8	20.4	21.8
eGFRcys: CKD-EPI 2012	-5	-6.3	-7	-9.5	15.2	16.1	19.8	19.6
eGFRcys: EKFC 2023	-3.5	-5.6	-7	-9.5	14.9	14.8	19.2	20.7
eGFRcys: CAPA	-4.2	-6.5	-8.1	-10.8	15.1	16.4	20.5	21.8
eGFRcr-cys: CKD-EPI 2012	2.6	0	-1.5	-1.8	11.4	12.6	15.1	16.7
eGFRcr-cys: CKD-EPI 2021	4.2	2.4	1.2	0.7	12.6	13.5	15.1	16.9
eGFRcr-cys: EKFC 2023	0.3	-2.3	-3.9	-4	10.7	12.5	16.3	19.1
eGFRcr-cys: LMR CAPA	-0.3	-3.8	-5.5	-6	9.9	12.2	16.7	18.5

Bias was estimated as median difference of eClcr or eGFR minus mGFR. IQR: interquartile range of the bias. CAPA: Caucasian, Asian, pediatric and adult; CKD-EPI: Chronic Kidney Disease Epidemiology Collaboration; eClcr: estimated creatinine clearance; eGFRcr: estimated GFR by creatinine; eGFRcys: estimated GFR by cystatin C; eGFRcr-cys: estimated GFR by creatinine and cystatin C; EKFC: European Kidney Function Consortium; LMR: Lund-Malmö Revised; mGFR: measured glomerular filtration rate (GFR) by single-point iohexol clearance. Green: small bias; Amber: medium bias; Red: large bias.

Table S12: Metastatic disease subgroups - Bias and Precision

	**Bias**			**Precision**		
	Median difference (mL/min)			IQR of the difference (mL/min)		
**eGFR**	Not metastatic	Metastatic	Unknown	Metastatic	Not metastatic	Unknown
eClcr: Cockcroft and Gault	-1.7	1	1.5	18.9	21.3	17.2
eGFRcr: CamGFRv2	-2.9	-1	-1.3	18.5	21.1	15.9
eGFRcr: CKD-EPI 2009	4.6	8	5.5	19.2	22.9	15.1
eGFRcr: CKD-EPI 2021	9.5	11.7	9.5	19.3	22.9	14.6
eGFRcr: EKFC 2021	0.3	2.1	1.9	18.3	19.6	13.6
eGFRcr: LMR	-2.2	1.3	0.4	18	20.8	14.3
eGFRcys: CKD-EPI 2012	-6.6	-8.3	-5.6	17.2	22.4	15.2
eGFRcys: EKFC 2023	-6.9	-7.8	-3.1	16.7	19.8	15.2
eGFRcys: CAPA	-7.8	-7.8	-4.9	17.7	21.9	15.6
eGFRcr-cys: CKD-EPI 2012	-0.5	-0.2	-0.5	13.7	17.1	10.8
eGFRcr-cys: CKD-EPI 2021	2.2	1.6	1.6	14.2	17.8	11.5
eGFRcr-cys: EKFC 2023	-2.7	-3.2	-0.9	14.6	15.3	11.1
eGFRcr-cys: LMR CAPA	-4.5	-3.7	-2.2	14.5	15.6	11.2

Bias was estimated as median difference of eClcr or eGFR minus mGFR. IQR: interquartile range of the bias. CAPA: Caucasian, Asian, pediatric and adult; CKD-EPI: Chronic Kidney Disease Epidemiology Collaboration; eClcr: estimated creatinine clearance; eGFRcr: estimated GFR by creatinine; eGFRcys: estimated GFR by cystatin C; eGFRcr-cys: estimated GFR by creatinine and cystatin C; EKFC: European Kidney Function Consortium; LMR: Lund-Malmö Revised; mGFR: measured glomerular filtration rate (GFR) by single-point iohexol clearance. Green: small bias; Amber: medium bias; Red: large bias.

Table S13: Subgroups: Proportion of individuals recommended an overdose of carboplatin compared to dose recommended by mGFR for AUC 5mg/mL/min of carboplatin

		Sex		BMI category (kg/m^2)				Metastatic disease		
eGFR	**Overall**	**Female**	**Male**	**<20**	**20 to <25**	**25 to <30**	**>=30**	**Not metastatic**	**Metastatic**	**Unknown**
eClcr: Cockcroft and Gault	10.4	12.6	9	12.8	10.5	8.7	12.2	9.7	13.3	9.4
eGFRcr: CamGFRv2	7.3	6.1	8	9.6	6.5	5.3	13	7.2	7.9	6.9
eGFRcr: CKD-EPI 2009	17.3	15.3	18.5	26.9	16.8	14.6	17.4	16.8	21	13.9
eGFRcr: CKD-EPI 2021	26.5	25.3	27.2	38.8	26.1	23.4	25.2	26.2	30.1	22.6
eGFRcr: EKFC 2021	11	9.2	12.1	17.8	10.1	9.5	11.9	10.7	13.3	8.7
eGFRcr: LMR	9.2	9.2	9.1	15.1	8.5	7.5	10.4	8.7	11.4	7.6
eGFRcys: CKD-EPI 2012	2.4	3.1	1.9	4.6	2.3	1.7	2.2	2.5	2.6	1.4
eGFRcys: EKFC 2023	1.7	2.1	1.4	3.2	1.7	1.4	0.7	1.5	2.1	1.7
eGFRcys: CAPA	2.5	4.5	1.3	4.6	2.4	2.2	2.2	2.3	4	1.4
eGFRcr-cys: CKD-EPI 2012	5	6.6	4	7.3	4.7	4.3	5.6	4.5	7.7	3.1
eGFRcr-cys: CKD-EPI 2021	7.7	9.4	6.7	11.4	8	6.4	7	7.5	9.8	5.6
eGFRcr-cys: EKFC 2023	3.1	3.6	2.8	6.8	2.8	2.5	3	2.8	4.7	2.4
eGFRcr-cys: LMR CAPA	2.9	4.3	2	4.6	2.2	3	3.7	2.5	4.9	1.7

AUC: area under the curve; eClcr CG: estimated creatinine clearance by Cockcroft and Gault; estimated GFR by creatinine; eGFRcys: estimated GFR by cystatin C; eGFRcr-cys: estimated GFR by creatinine and cystatin C; EKFC: European Kidney Function Consortium; mGFR: measured glomerular filtration rate (GFR) by single-point iohexol clearance. Green: $\leq$5%; Amber: >5-10%; Red: >10%.

Table S14: Subgroups: Proportion of individuals recommended an underdose of carboplatin compared to dose recommended by mGFR for AUC 5mg/mL/min of carboplatin

eGFR	Overall	Sex		BMI category (kg/m^2)				Metastatic disease		
		**Female**	**Male**	**<20**	**20 to <25**	**25 to <30**	**>=30**	**Not metastatic**	**Metastatic**	**Unknown**
eClcr: Cockcroft and Gault	6.1	6.4	5.9	6.4	5.3	7.8	4.4	5.9	6.8	5.9
eGFRcr: CamGFRv2	6	7.4	5.2	1.8	6.7	7.6	3.7	5.5	8.2	5.6
eGFRcr: CKD-EPI 2009	1.8	1.7	1.8	0.5	1.5	2.3	2.2	1.6	2.1	2.1
eGFRcr: CKD-EPI 2021	0.8	0.8	0.8	0.5	0.8	1.1	0.4	0.5	1.4	1.4
eGFRcr: EKFC 2021	3.1	3.6	2.8	0.9	2.2	4.8	3.7	2.9	3.7	3.1
eGFRcr: LMR	4.8	4.2	5.1	0.9	3.9	7.3	4.4	5	4.7	3.8
eGFRcys: CKD-EPI 2012	18	16.8	18.9	18.3	17	17.7	22.2	16.1	23.4	18.8
eGFRcys: EKFC 2023	13.9	10	16.3	14.6	13.1	14	15.6	12.1	20.1	12.5
eGFRcys: CAPA	18.1	13.2	21.1	18.3	17	17.9	22.2	16.4	24.3	16.3
eGFRcr-cys: CKD-EPI 2012	3.7	4	3.5	1.4	3.1	4.7	5.2	2.6	5.4	6.2
eGFRcr-cys: CKD-EPI 2021	2.9	3.1	2.7	1.8	2.4	3.1	4.4	2	4	4.9
eGFRcr-cys: EKFC 2023	3.5	2.9	3.9	1.4	3	4.5	4.4	2.7	5.1	4.5
eGFRcr-cys: LMR CAPA	4.8	3.6	5.4	0.9	3.9	6.1	7.4	4	6.1	5.9

AUC: area under the curve; eClcr CG: estimated creatinine clearance by Cockcroft and Gault; estimated GFR by creatinine; eGFRcys: estimated GFR by cystatin C; eGFRcr-cys: estimated GFR by creatinine and cystatin C; EKFC: European Kidney Function Consortium; mGFR: measured glomerular filtration rate (GFR) by single-point iohexol clearance. Green: $\leq$5%; Amber: >5-10%; Red: >10%.

Supplementary Figures

Figure S1: Bland-Altman plots for agreement between mGFR and eGFR

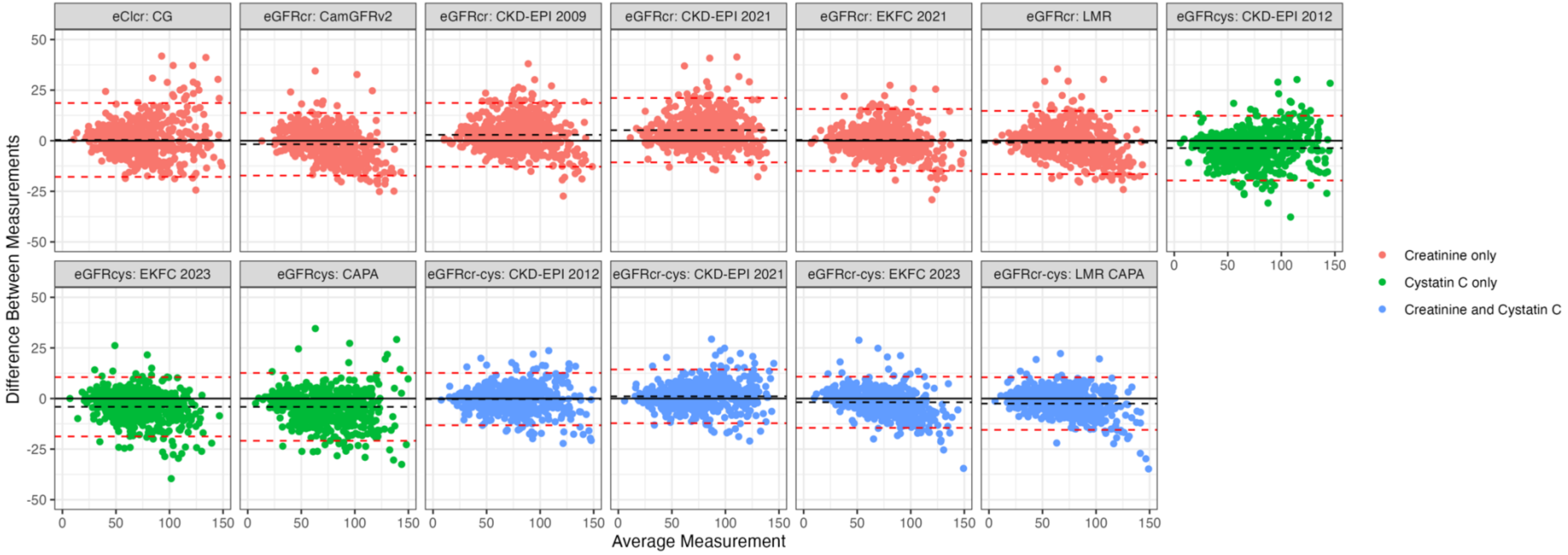

A random 25% of values are presented to prevent clutter. Dotted black line represents the mean bias. Dotted red lines represent values ± 1.96 standard deviations from the mean. CAPA: Caucasian, Asian, pediatric and adult; CKD-EPI: Chronic Kidney Disease Epidemiology Collaboration; CG: Cockcroft and Gault; eClcr: estimated creatinine clearance; eGFRcr: estimated GFR by serum creatinine; eGFRcys: estimated GFR by serum cystatin C; eGFRcr-cys: estimated GFR by serum creatinine and cystatin C; EKFC: European Kidney Function Consortium; LMR: Lund-Malmö Revised; mGFR: measured glomerular filtration rate (GFR) by single-point iohexol clearance.

Figure S2: Subgroups: Plots of recommended dosing of carboplatin compared to mGFR

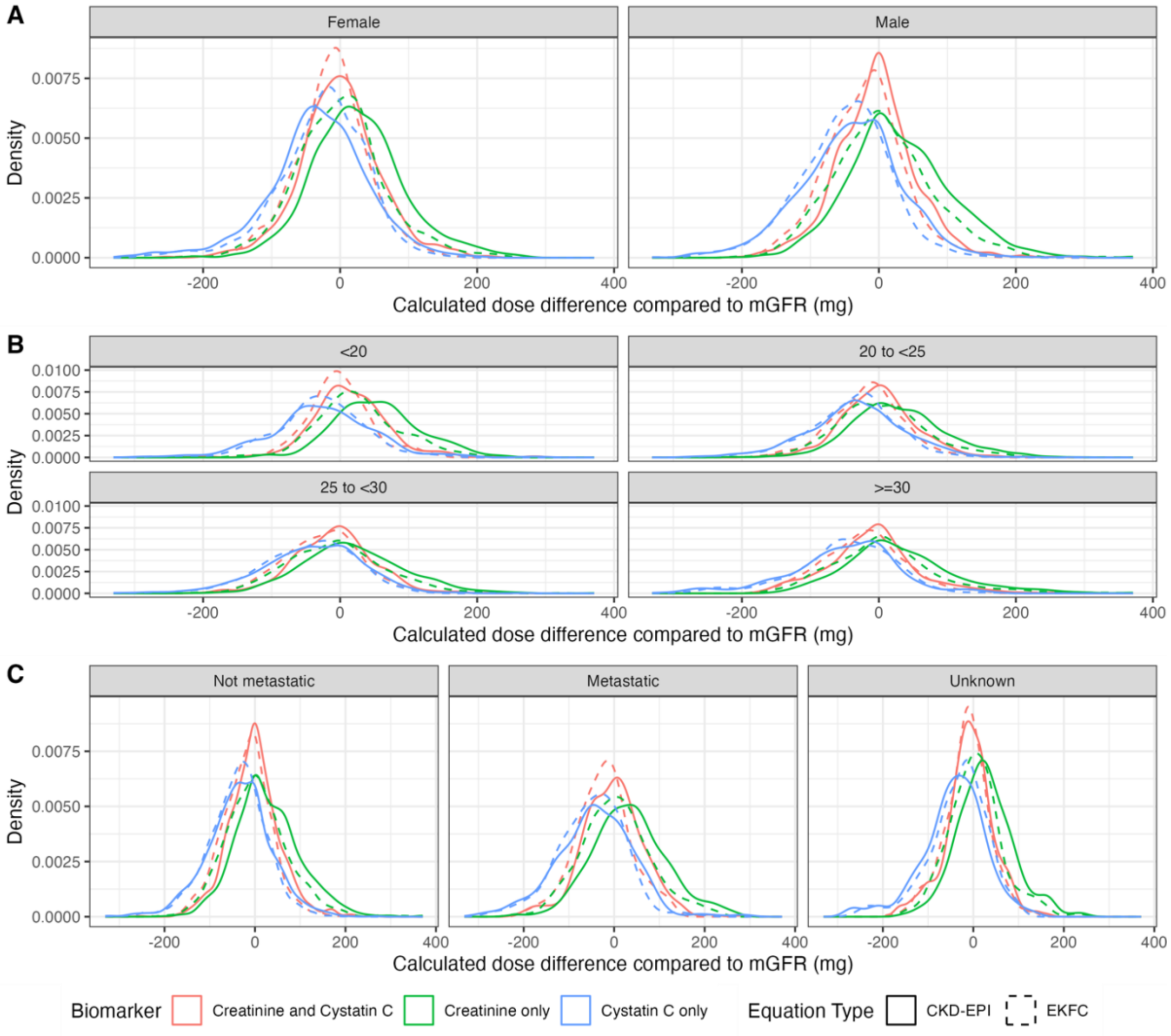

Density plots showing the difference in recommended dose of carboplatin (to achieve AUC 5mg/mL/min) calculated by eClcr, eGFRcr, eGFRcys and eGFRcr-cys compared to mGFR in subgroups by: (A) Sex; (B) Body mass index category (kg/m^2); (C) Metastatic disease status. Positive values indicate a higher dose than was calculated by mGFR; negative values represent a lower dose than was calculated by mGFR. eClcr: estimated creatinine clearance; eGFRcr: estimated GFR by creatinine; eGFRcys: estimated GFR by cystatin C; eGFRcr-cys: estimated GFR by creatinine and cystatin C; EKFC: European Kidney Function Consortium; mGFR: measured glomerular filtration rate (GFR) by single-point iohexol clearance.
